# Microcalorimetry reveals multi-state thermal denaturation of *G. stearothermophilus* tryptophanyl-tRNA synthetase

**DOI:** 10.1063/4.0000181

**Published:** 2023-07-18

**Authors:** Srinivas Niranj Chandrasekaran, Jhuma Das, Nikolay V. Dokholyan, Charles W. Carter

**Affiliations:** 1 Imaging Platform, Broad Institute of MIT and Harvard, Cambridge, Massachusetts 02142, USA; 2 Cystic Fibrosis and Pulmonary Diseases Research and Treatment Center, University of North Carolina at Chapel Hill, Chapel Hill, North Carolina 27599, USA; 3 Department of Pharmacology and Biochemistry and Molecular Biology, Penn State College of Medicine, Hershey, Pennsylvania 17033, USA; 4 Department of Biophysics and Biochemistry, University of North Carolina at Chapel Hill, Chapel Hill, North Carolina 27599, USA

## Abstract

Mechanistic studies of *Geobacillus stearothermophilus* tryptophanyl-tRNA synthetase (TrpRS) afford an unusually detailed description—the escapement mechanism—for the distinct steps coupling catalysis to domain motion, efficiently converting the free energy of ATP hydrolysis into biologically useful alternative forms of information and work. Further elucidation of the escapement mechanism requires understanding thermodynamic linkages between domain configuration and conformational stability. To that end, we compare experimental thermal melting of fully liganded and apo TrpRS with a computational simulation of the melting of its fully liganded form. The simulation also provides important structural cameos at successively higher temperatures, enabling more confident interpretation. Experimental and simulated melting both proceed through a succession of three transitions at successively higher temperature. The low-temperature transition occurs at approximately the growth temperature of the organism and so may be functionally relevant but remains too subtle to characterize structurally. Structural metrics from the simulation imply that the two higher-temperature transitions entail forming a molten globular state followed by unfolding of secondary structures. Ligands that stabilize the enzyme in a pre-transition (PreTS) state compress the temperature range over which these transitions occur and sharpen the transitions to the molten globule and fully denatured states, while broadening the low-temperature transition. The experimental enthalpy changes provide a key parameter necessary to convert changes in melting temperature of combinatorial mutants into mutationally induced conformational free energy changes. The TrpRS urzyme, an excerpted model representing an early ancestral form, containing virtually the entire catalytic apparatus, remains largely intact at the highest simulated temperatures.

## INTRODUCTION

Mechanistic studies of *B. stearothermophilus* tryptophanyl-tRNA synthetase (TrpRS) identified two subtle, and apparently contradictory, ways in which differential conformational stability affects its catalytic cycle.^1–4^ Catalytic assist by Mg^2+^ ion, on the one hand, depends entirely on its coupling to domain motions.^3,5,6^ On the other hand, the sign of the free energy change for the catalytic conformational change is positive in the presence of the pyrophosphate product, PPi, and negative only in its absence.^7^ That deepened understanding, in turn, provides a novel perspective on how biology appears to circumvent the second law of thermodynamics.^4,8,9^

The former effect couples assembly of the fully active catalytic apparatus tightly to domain motion; the latter ensures that domain motion requires PPi release, thereby coupling the conformational change reciprocally^10^ to the chemistry of ATP utilization. This novel escapement mechanism^3,4^ ensures efficient vectorial utilization of ATP^11,12^ and is thus a potentially general model for energetic coupling in a broader range of enzymes that transduce NTP hydrolysis free energy.^13,14^ Neither front nor back-end gating has been analyzed in detail for any other transducing NTPases, although just as the TrpRS catalytic domain movement is triggered by PPi release, the myosin power stroke appears to be triggered by phosphate release.^13,14^ The back-end gating may, thus, be common to many NTPases.

Key to the escapement mechanism are conformational stability changes induced, in turn, by the succession of bound ligands, as chemical events proceed.^2^ Untangling different levels of energetic coupling that produce the contradictory gating effects necessarily entails measuring how these stability changes arise from differential ligand binding. That task is facilitated by a set of combinatorial mutants within a packing motif called the D1 switch that mediates shear developed during TrpRS domain motion.^1,15^ The D1switch consists of 6–7 residues near the C-terminus of the Rossmann fold N-terminal α-helix that bind the preceding and following β-strands to that helix. It is one of the most widely conserved motifs in the proteome^16^ and central to this work.

We stumbled on the D1 switch motif when we attempted to identify residues whose nearest neighbors changed as TrpRS passed through its conformational trajectory during catalysis.^17^ Delaunay tessellation decomposes protein packing interactions uniquely into tetrahedra of nearest-neighbor side chains.^18,19^ TrpRS crystal structures revealed that the vast majority of the ∼300 tetrahedra lie within domains that move as rigid bodies and are, thus, structurally invariant. Only about a dozen tetrahedra are structurally dynamic. A series of papers using the PATH algorithm to map the conformational changes during catalysis showed that repacking of aromatic residues in the D1 switch motif—F26, Y33, and F37—constitutes the rate-limiting step in assembling the active site for catalysis upon binding tryptophan and ATP.^1,7,20^

Rosetta^21^ identified specific mutations of the three aromatic D1 residues plus I4 that should stabilize the excited Pre-TS state relative to the two ground states—Open and Products—on either side.^17^ Combinatorial mutagenesis of those residues showed that repacking is coupled by ∼–5.5 kcal/mol to the active-site Mg^2+^ ion. It accounts for the contribution of the metal to transition-state stabilization. A subsequent modular thermodynamic cycle^22^ implicated an untwisting domain motion to catalysis by the same coupling free energy. Those studies linked dynamic side chain re-packing in the D1 switch alternately to domain motion and to Mg^2+^-dependent rate acceleration of amino-acid activation. Recent studies of a related thermodynamic cycle measuring the energetic coupling between the HVGH and KMSKS catalytic signatures in the larger leucyl-tRNA synthetase^23^ provide complementary evidence that such coupling is common to all class I aaRS. The ensemble of structural, kinetic, and computational studies on the D1 switch afforded a uniquely consistent set of cross-validating intercorrelations (see Fig. 7 in Ref. 20). Those correlations underlie the TrpRS escapement mechanism and so strongly motivate the present analysis of TrpRS melting behavior.

In this report, we use differential scanning calorimetry (DSC) and computational simulation to compare the melting behavior of fully liganded TrpRS bound to ATP and a non-reactive tryptophan analog stabilizing the crystallographic pre-transition conformational state (1MAU^24^) with that of the unliganded apo form (1D2R^25^). Given the differences between experimental (TrpRS dimer; Cp; heat capacity at constant pressure) and computational (TrpRS monomer; Cv; heat capacity at constant volume) methods, there is surprising consistency between the two methods. Moreover, the computational simulations also allow comparison of two structural metrics—the radius of gyration, R_g_, and α-helical content—that facilitate interpretation. Our principal observations are as follows:

(i) Both liganded and apo TrpRS denature via a three-state process involving a molten globular intermediate that forms at temperatures ∼8° lower than those required to melt α-helical secondary structures.
(ii) A broad, unidentified and substantial heat capacity change occurs at approximately the temperature at which the source organism grows optimally and precedes the formation of the molten globule.
(iii) A computational free energy surface of a liganded TrpRS monomer exhibits similar multi-step experimental heat capacity changes. That simulation is reversible by definition, because the isolated monomer cannot aggregate. It also furnishes valuable structural metrics and cameo snapshots that aid interpretation of the melting behavior.
(iv) The catalytic core known as the urzyme, an experimentally validated model for the ancestral form of class I aminoacyl-tRNA synthetases, containing only the 130 residues necessary to frame the active site, is the last remaining folded structure at high temperatures in the computed free energy surface. Preservation of the tertiary packing of D1 residues at high temperature appears to stabilize the N-terminal α-helix, which has only weak helical propensity.

An accompanying paper^26^ describes melting curves obtained by differential scanning fluorometry and far UV circular dichroism for unliganded native and 15 combinatorial TrpRS D1 switch variants. Analysis of unliganded TrpRS described here provide a solid context in which to identify the intrinsic and higher-order structural interactions that allow the D1 switch residues to stabilize or destabilize different conformational states along the structural reaction profile, hence their functional behavior. Our goal is to establish a basis for pursuing two non-trivial longer-range questions: (i) Can we exploit the mutant proteins to correlate stability changes of all three conformations with the steady-state kinetic properties measured previously?^3^ (ii) How does coupling between the four mutated residues change along the conformational reaction path, allowing them to exert their highly cooperative contribution to the escapement mechanism?^4^

## METHODS

### Differential scanning calorimetry

TrpRS solutions (2 *μ*M) were dialyzed overnight against either buffer alone [20 mM HEPES, 50 mM NaCl_2_ (pH 7.0)] of the same buffer plus tryptophanamide (50 *μ*M) and ATP (10 mM). In each case, we reserved the dialysate for use as a blank in calorimetry. We loaded 500 *μ*l of each of the four solutions (sample plus blank, liganded vs apo) into the chamber of a TA Instruments Nano DSC 250 microcalorimeter. Thermal scans were performed at 2.98 atm from 10 to 95 °C in 5 min intervals. Data from the thermal scans were processed using the TA Nanoanalyze software, v. 3.12 (https://www.tainstruments.com/itcrun-dscrun-nanoanalyze-software/) to subtract the blank profile, normalize the net heat capacity curves, select baselines, and identify peaks. We identified and integrated heat capacity peaks in the profiles using the Voigt Gaussian/Lorentzian hybrid given an onset temperature (Tonset), which gave superior local fit to all curves.

### Replica exchange DMD simulations

We make use here of an extended analysis of the free energy surface connecting the pre-transition state (PreTS) and products (Products) conformational states. The replica exchange algorithm efficiently samples the temperature-dependent conformational free energy landscape of a macromolecule by simulating replicas of the equilibrium state at different temperatures and, at predefined time points, exchanging the structures at different temperatures if the difference in their energies is within a threshold. It is reversible by definition because the monomer cannot aggregate.

Our original purpose was to find an independent estimate of the conformational transition-state structure that we could compare with that produced by a minimum action path algorithm.^7^ To that end, we projected the simulated trajectory onto the two dominant principal components of the motion to give a 3D function whose z-axis was conformational free energy. That surface was used to map the location of the conformational transition states separating the Pre-transition and Product state conformations to the stationary point of the conformational transition path computed using a minimum action path algorithm and which turned out in excellent agreement with the position of the global saddle point separating the PreTS and Products states in the replica exchange simulation.

We implemented the replica exchange algorithm using the REX/DMD suite^27–30^ that uses Discrete Molecular Dynamics^28,30–32^ to simulate replicas at 24 temperatures ranging from ∼ 175 to ∼ 405 K separated by a constant interval. DMD approximates atomic interactions by multistep square well potentials^27,33^ and uses an Andersen Thermostat to regulate system temperature, while the Lazaridis–Karplus implicit solvation model^34^ accounts for the solvation energy. Simulations were set up with the Product state structure, 1I6L, with the PreTS state structure, 1MAU which appears to be the least stable of the crystal structures.^17^

We excised the terminal amino acid (R328) from the structures as it does not appear in most crystal structures. Also, since DMD force field does not include parameters for Mg^2+^, we replaced it with Zn^2+^. The two metals have almost exactly the same ionic radii and hydration energies, and they behave almost identically in molecular simulations with empirical force fields.^35^ Moreover, Zn^2+^ ion parametrizations prove to be the most similar to those of Mg^2+^ in potential of mean force profiles used to model ionic interactions with nucleic acids.^36^ For these reasons, we do not expect these changes to affect the DMD of the system in a significant manner. We applied a harmonic constraint (well width = ±1 Å) between ligand atoms and the binding pocket within 3.5 Å of each other to retain ligands in their binding pockets. We applied another weak harmonic constraint (well width = ±2 Å) to atoms which uniquely form native contacts (8 Å), in either 1I6L or 1MAU, in order to sample conformational spaces adjacent to the two boundary states. Simulations at each temperature ran for 3 × 10^6^ steps ( ∼ 150 ns). Omitting the time to equilibrate (initial 500 000 steps), snapshots were generated every 1000 steps for a total of 2500 snapshots which were then used in all the analyses.

Coordinates from the trajectory furnished a variety of useful metrics to aide interpretation of the melting behavior. The WHAM—weighted histogram analysis method—algorithm enable computation of the specific heat capacity, $C_{v}$,^37^ from the energies of structures generated at different temperatures. The atomic coordinates of the TrpRS monomer enable computation of the average radius of gyration, R_g_ from the mean structures at each temperature. Similarly, we used the DSSP algorithm^38,39^ as implemented in Pymol^40^ with default options to compute the fraction of α-helical residues for each averaged structure.

## RESULTS AND DISCUSSION

### TrpRS melts by a multi-step, ligand-dependent process

**FIG. 1.**
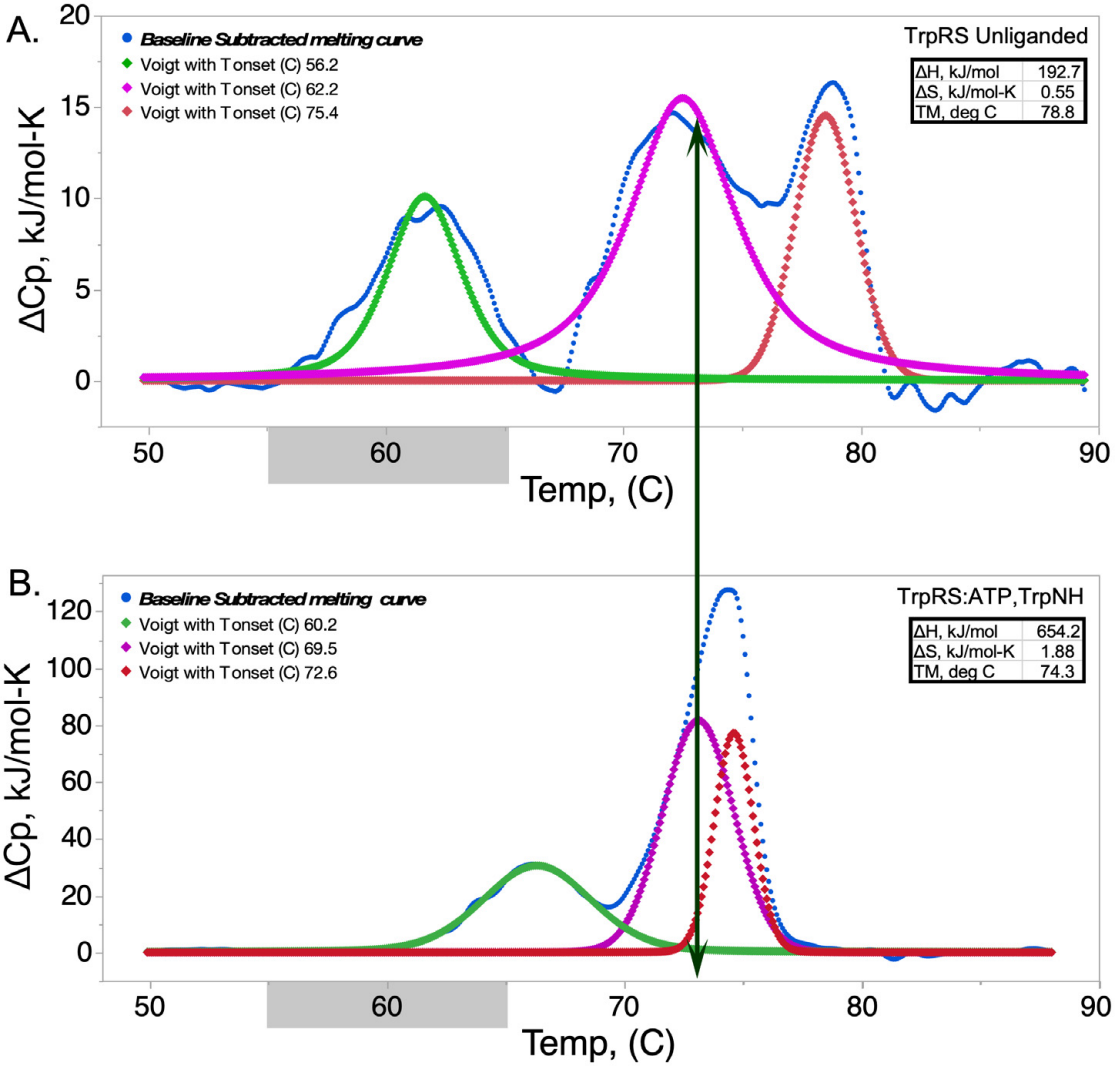
Heat capacity changes in the melting behavior of apo (a) and fully liganded (b) *Bacillus stearothermophilus* tryptophanyl-tRNA synthetase. Differential scanning calorimetry was carried out and processed using TA NanoAnalyze software as described in Methods section. The vertical arrow is to facilitate alignment of two peaks related to the breakdown of non-polar packing (magenta) and secondary structure (red). The gray bars along the x axis indicate the normal growth temperature range for *Geobacillus stearothermophilus.*

Experimental heat capacity changes during TrpRS thermal melting exhibit complex behavior with clear differences between apo [Fig. 1(a)] and liganded [Fig. 1(b)] forms. Binding of tryptophanamide and ATP lead to an inhibited state we have termed the pre-transition-state (Pre-TS) conformation because the tryptophanamide and ATP ligands trap it just prior to the chemical step in tryptophan activation. Crystal structures of the apo and Pre-TS states differ substantially^24,25,41^ as the anticodon-binding domain rotates toward the catalytic domain by ∼8° and twists by ∼3.5° in the PreTS state. The different melting curves must reflect these structural differences.

Melting occurs in three distinguishable stages: initial (T_onset_ = 56, 60 °C), intermediate (T_onset_ = 62,69 C) with a major change in heat capacity, and terminating (T_onset_ = 75, 73 °C). Pre-TS TrpRS melting is also significantly more cooperative with all changes occurring between 62 and 73 °C], compared to 56 and 82 °C.

Curiously, although ligand binding normally stabilizes protein structures against thermal melting, the overall melting of the apo form occurs at higher temperature (79 vs 74 °C). That observation confirms previous estimates, derived from nucleotide-ligand binding affinity changes between different conformations and from comparing statistical potentials derived from side chain packing analysis which led us to conclude that the Pre-TS state was significantly destabilized by the conformation induced prior to catalysis.^17^

### Multi-step experimental melting behavior matches that observed in a computational free energy surface of a liganded TrpRS monomer

**FIG. 2.**
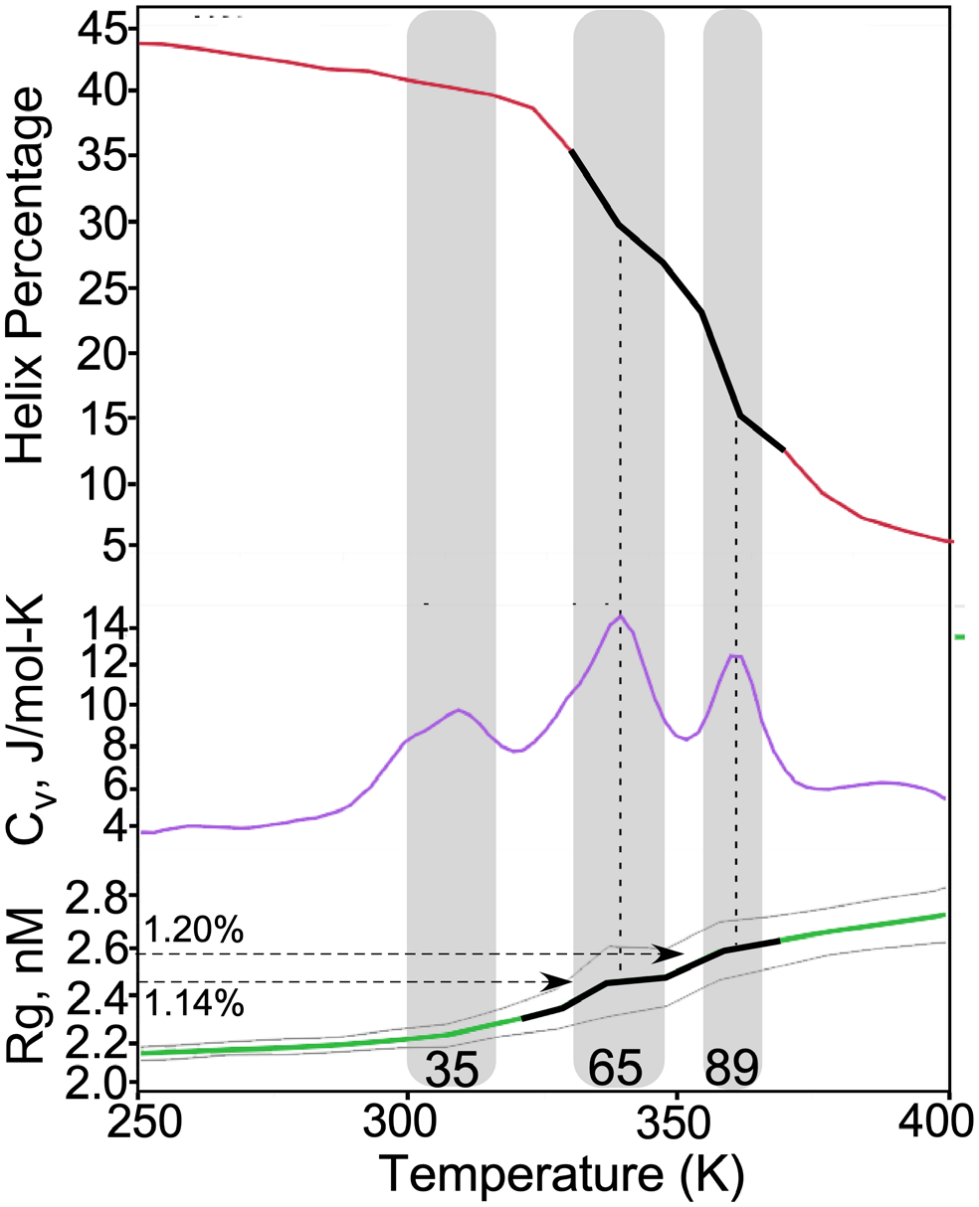
Computational traces of structural and thermodynamic changes associated with fully liganded monomeric TrpRS thermal denaturation. Gray shading highlights successive maxima in the specific heat capacity, C_v_, (blue curve). Temperatures in °C are included above the x axis to facilitate comparison with the experimental melting curves in Fig. 1. Each transition corresponds to increases in volume, as indicated by the radius of gyration (R_g_, green curve, bounded by its uncertainties in gray). The 14% increase in R_g_ in the second transition corresponds to that (15–20%) expected from formation of a molten globule.^44^ Similarly, the loss in helical content (red curve) occurs in two stages, the more dramatic of which coincides with the second transition with an R_g_ value increased by 20%. The two, principal, symmetric peaks occur at temperatures corresponding to those measured by Thermofluor (65 vs 69 °C) and ellipticity (89 vs 80 °C). Vertical dashed lines align discontinuities in both structural parameters (black lines highlight transitions of interest) with the stationary points of the C_v_ trace.

To validate the thermodynamic interpretations of TrpRS melting behavior, we take advantage here of detailed correspondences—and differences—between the experimental melting curves (Fig. 1) and those derived from a computational temperature-dependent conformational free energy surface of the liganded, wild-type TrpRS monomer PreTS complex. That surface (Fig. 2) is reversible by definition,^42^ because the isolated monomer cannot aggregate. It was constructed previously using Replica Exchange Discrete Molecular Dynamics (REX/DMD^29,42,43^) simulations to confirm the computational transition states identified by a minimum action path analysis.^1,7,20^

Figure 2 shows heat capacity changes, $C_{v}$, as a function of temperature. That plot shares important characteristics with the experimental melting curves. All three show three main transitions; they have the same relative peak heights, integrated areas, and relative widths. They differ only in the temperatures at which they occur. We discuss possible reasons for this discrepancy further in the subsequent section discussing influences on the melting.

Thus, the correspondence between experimental Thermofluor and CD melting curves (Fig. 1) and computational occurrences of these two transition events (Fig. 2) is significantly detailed.

### Structural details associated with the simulated melting facilitate interpretation

Structural metrics—radius of gyration, R_g_, and percentage helix—computed from the mean coordinates of the DMD trajectories accompany the simulated heat capacity changes obtained during the DMD simulation. Temperature-dependences of both metrics are consistent with the maxima in the $C_{v}\;\text{plot}.$ Heat capacity maxima are stationary points where the protein absorbs and loses excess heat from and to the system in equal amounts, as it undergoes conformation changes, so heat capacity changes transiently remain constant with respect to system temperature. In keeping with stationary behavior, local maxima in the $C_{v}$ traces for the two major transitions, coincide with discontinuities in the slopes of both structural metrics, each changing faster before the $C_{v}$ maximum and more slowly afterward (black tracings, Fig. 2).

The average R_g_ increases by 14% during the second transition, corresponding to the increase expected for a molten globule transition (15%).^44^ R_g_ increases again by 20% at 87 °C. At the 67 °C transition temperature for the second peak of the specific heat capacity curve, 70% of the helices present in the native protein remain intact. At 87 °C, the third transition temperature, only 28% of the native helices remain intact. Each heat capacity peak, therefore, corresponds to a structurally different transition in both metrics and thus to a different unfolding process.

The initial peak in the $C_{v}$ trace is broader, has a smaller amplitude, and involves minimal changes in either structural metric. It is evident mainly because it initiates the increasing variance of R_g_, which occurs almost exclusively with the formation of the molten globule. As 60 °C coincides with the optimal growth temperature for *Geobacillus stearothermophilus*, it is possible that this local maximum in $C_{v}$ has biological significance. However, temperature-dependent activity curves for native TrpRS^45^ are not informative on this point.

### The dimer interface and the presence of active-site ligands strongly influence TrpRS melting

**FIG. 3.**
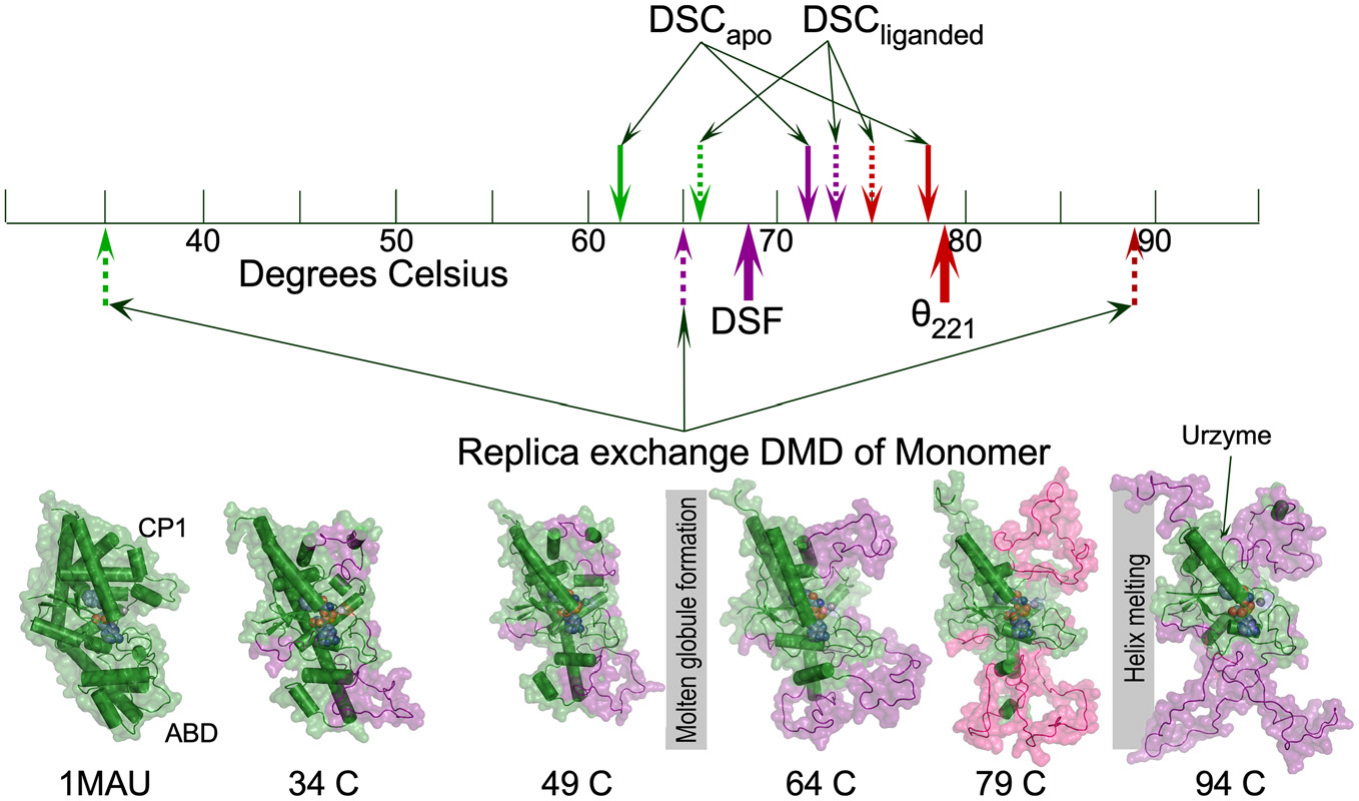
TrpRS melting transitions. Experimental differential scanning calorimetry is above and replica exchange DMD simulation below the temperature scale. Dashed lines indicate fully liganded molecules. Computational traces are for the monomer, not the dimer as above the scale. Bottom row shows Pymol cartoons of mean structures observed for the ensemble of 24 replicas at the indicated temperatures. These temperature-dependent transitions in the overall trajectory help guide the interpretations of the melting events. We identified native secondary structures (forest green) using the DSSP criterion used to identify native α-helices in Fig. 2; denatured segments are deep purple. Gray vertical bars indicate molten globular and helix-melting transitions.

Figure 3 compares transitions in experimental and simulated melting curves. The computational melting transitions distribute substantially more widely than the experimental ones in Fig. 1. The dimer interface buries ∼2160 Å^2^ of surface area in the PreTS state. That value suggests a rather low dimer dissociation constant 10^−8^ < K_D_ < 10^−6^.^46^ We previously used scanning force microscopy to count monomers and dimers at successively higher dilutions to measure the strength of the dimer interface.^47^ Those estimates, 6.6 ± 4  × 10^−9^ for unliganded and 1.1 ± 1 × 10^−8^ M for a complex with a non-hydrolysable aminoacyl-5′-adenylate product analog, are at the lower end of that range. Thus, considerable binding free energy is bound into the dimer dissociation constant, which probably induces a considerable difference between the melting behavior of monomer and dimer. Although we have no ready means to verify the assumption, we reason that much of the difference between the three melting temperatures in Fig. 3 may arise because we simulated the TrpRS monomer, not the dimer, and that the dimer interface also compresses the separation between transitions.

Consistent with that assumption, Pre-TS ligands compress the experimental traces still further to a narrower temperature range over which the three melting events occur (dashed lines above the scale vs solid lines above the temperature scale). Simulation of the monomer shows the same three melting events seen in the DSC of the dimer, but they are separated much more widely in temperature. Structural cartoons through the middle and high temperature thermal events both entail significant volume increases, supporting identification of the middle transition as formation of a molten globule.

We assembled structural snapshots over the course of the exchange trajectory from each replica at given temperatures, noted in Fig. 3. The average structures at each temperature were analyzed for native helical content using DSSP as indicated in Fig. 1. Notably, melting is localized to both the connecting peptide 1 (CP1) and anticodon-binding (ABD) domains (deep purple surfaces). The transition at 65 °C can reasonably be assigned to molten globule formation because tertiary structures of those two domains are expanded, but not unfolded.

### The TrpRS urzyme appears to unfold last

**FIG. 4.**
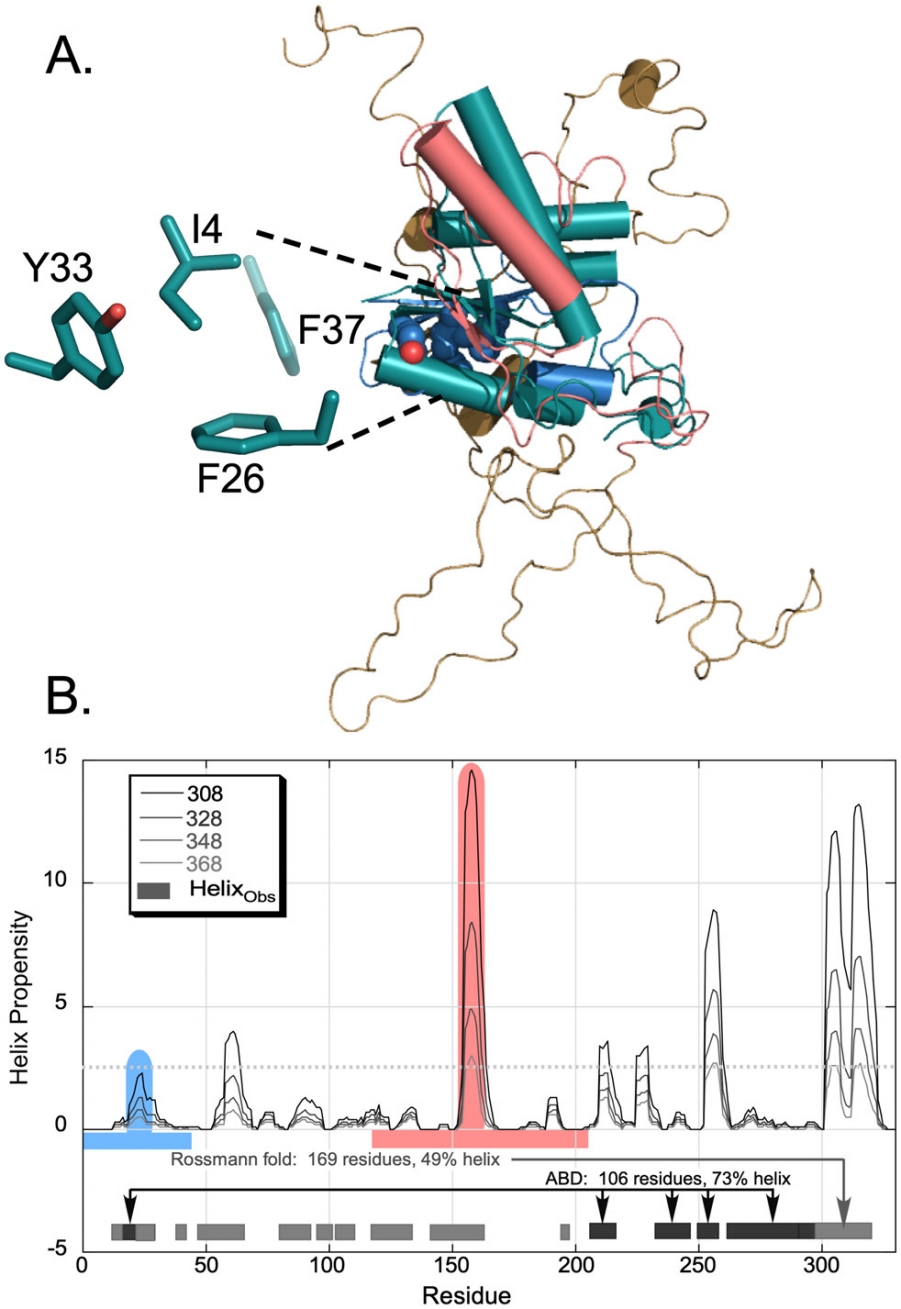
The TrpRS catalytic apparatus denatures last. (a) The TrpRS urzyme (teal^49,51^) is superimposed on the averaged structure from the ensemble at 94 °C (sand, sky, and salmon). The first crossover of the urzyme is colored sky, and the second is colored Salmon in keeping with the colors in (b). Teal sticks are the four D1 switch residues. The urzyme remains largely intact whereas most of the rest of the monomer is unfolded. (b) TrpRS helix propensities computed using the Agadir server^52^ for different temperatures. Only three of the helices observed in the TrpRS structure (residues 154–166; residues 253–261; and residues 303–324) have peak propensities exceeding 2.5 (dashed line) at the highest temperature used in our experiments (368 K). The Rossmann fold (gray) and anticodon-binding (ABD; black) domains are interleaved; the first helix participates in both domains, as its N-terminus moves as a rigid body with the ABD. This interleaving likely contributes to the high cooperativity of the helix melting transition monitored by θ_221._^26^ The two colored segments together comprise the urzyme.

Ding *et al.*^48^ suggested the persistence of a core 3D structure in unfolded forms of many proteins. Remarkably, for TrpRS, that core, Fig. 4, coincides closely with its urzyme (ur = primordial, authentic, original + enzyme^49^), a model for the ancestral enzyme that itself appears to be a catalytically active molten globule.^50^ The TrpRS urzyme was constructed by fusing two disjoint segments with similar secondary structures—N- and C-terminal β-α-β crossover connections.^51^ One of the core helices that remains intact at 94 °C in the computational profile is the long helix (154–165) from the C-terminal segment (salmon). The sequence of that segment has an unusually high fundamental helix propensity as determined by the physics-based and unbiased database-independent Agadir algorithm.^52^ In contrast, the helix in the N-terminal segment, has a far lower propensity, but its first eight residues nevertheless remain helical at 368 K, suggesting that extrinsic forces sustain it. Further discussion in the accompanying paper^26^ considers what these extrinsic forces might be.

Hilvert's work with chorismate mutase^53,54^ identified an efficient, potentially widespread coupling between folding and catalysis—i.e., high transition-state affinity. In light of those findings, it is notable that the last part of the TrpRS monomer to melt in the REX/DMD free energy simulations closely approximates the TrpRS urzyme, a construct studied as a model for the evolution of aminoacyl-tRNA synthetases (Fig. 4). At 94 °C, the CP1 and ABD domains, as well as the long-C-terminal alpha helix are all fully unfolded. Thus, the modules that remain intact at the highest temperatures are also likely the most ancient part of the protein which, when excerpted, retains a full range of catalytic activities.^49,51,55^ The structural integrity of the TrpRS urzyme at high temperature is consistent with the experimental observation that the urzyme itself seems to function as a molten globule,^50^ whose only well-folded state is its complex with the transition state for amino acid activation.

## CONCLUSIONS

*G. stearothermophilus* tryptophanyl-tRNA synthetase is notable because multidisciplinary mechanistic studies have worked out reciprocally coupled gates making catalysis conditional to domain motion and, paradoxically, making domain motion conditional on catalysis. Maximizing the efficiency of converting the free energy of ATP hydrolysis to useful forms of work and information requires both gating functions.^4,10^ This paper describes thermal melting studies pursuant to attributing thermodynamic aspects of gating to specific amino acid residues in the D1 switch, a molecular switching motif that imposes multi-state behavior. The accompanying paper^26^ outlines a paradigm for using high throughput differential scanning fluorimetry to derive thermodynamic estimates for inter-residue energetic coupling, potentially in a ligand-dependent framework.

## Data Availability

The data that support the findings of this study are available from the corresponding author upon reasonable request.

## References

[1] C. W. Carter, Jr. , S. N. Chandrasekaran , V. Weinreb , L. Li , and T. Williams , “ Combining multi-mutant and modular thermodynamic cycles to measure energetic coupling networks in enzyme catalysis,” Struct. Dyn. 4, 032101 (2017). 10.1063/1.4974218 28191480 PMC5272822

[2] C. W. Carter, Jr. , “ High-dimensional mutant and modular thermodynamic cycles, molecular switching, and free energy transduction,” Annu. Rev. Biophys. 46, 433–453 (2017). 10.1146/annurev-biophys-070816-033811 28375734 PMC5472044

[3] V. Weinreb , L. Li , and C. W. Carter, Jr. , “ A master switch couples Mg^2+^-assisted catalysis to domain motion in *B. stearothermophilus* tryptophanyl-tRNA synthetase,” Structure 20, 128–138 (2012). 10.1016/j.str.2011.10.020 22244762 PMC3259537

[4] C. W. Carter, Jr. , “ Escapement mechanisms: Efficient free energy transduction by reciprocally-coupled gating,” Proteins 88, 710–717 (2019). 10.1002/prot.25856 31743491 PMC9486351

[5] V. Weinreb , L. Li , L. S. Kaguni , C. L. Campbell , and C. W. Carter, Jr. , “ Mg^2+^-assisted catalysis by *B. stearothermophilus* TrpRS is promoted by allosteric effects,” Structure 17, 952–964 (2009). 10.1016/j.str.2009.05.007 19604475 PMC2821082

[6] V. Weinreb and C. W. Carter, Jr. , “ Mg^2+^-free *B. stearothermophilus* tryptophanyl-tRNA synthetase activates tryptophan with a major fraction of the overall rate enhancement,” J. Am. Chem. Soc. 130, 1488–1494 (2008). 10.1021/ja076557x 18173270 PMC2826132

[7] S. N. Chandrasekaran , J. Das , N. V. Dokholyan , and C. W. Carter, Jr. , “ A modified PATH algorithm rapidly generates transition states comparable to those found by other well established algorithms,” Struct. Dyn. 3, 012101 (2016). 10.1063/1.4941599 26958584 PMC4769271

[8] E. Branscomb , T. Biancalani , N. Goldenfeld , and M. Russell , “ Escapement mechanisms and the conversion of disequilibria; the engines of creation,” Phys. Rep. 677, 1–60 (2017). 10.1016/j.physrep.2017.02.001

[9] E. Branscomb and M. J. Russell , “ Frankenstein or a submarine alkaline vent: Who is responsible for abiogenesis? Part 1: What is life–that it might create itself?,” BioEssays 40, 1700179 (2018). 10.1002/bies.201700179 29870581

[10] C. W. Carter, Jr. and P. R. Wills , “ Reciprocally-coupled gating: Strange loops in bioenergetics, genetics, and catalysis,” Biomolecules 11, 265 (2021). 10.3390/biom11020265 33670192 PMC7916928

[11] W. P. Jencks , “ Reaction mechanisms, catalysis, and movement,” Prot. Sci. 3, 2459–2464 (1994). 10.1002/pro.5560031232 PMC2142781 7757002

[12] W. P. Jencks , “ Utilization of binding energy and coupling rules for active transport and other coupled vectorial processes,” Methods Enzymol. 171, 145–164 (1989). 10.1016/S0076-6879(89)71010-7 2531833

[13] T. Kraft , E. Mählmann , T. Mattei , and B. Brenner , “ Initiation of the power stroke in muscle: Insights from the phosphate analog AlF4,” Proc. Natl. Acad. Sci. U. S. A. 102, 13861–13866 (2005). 10.1073/pnas.0504026102 16174728 PMC1236544

[14] A. Houdusse and H. L. Sweeney , “ How myosin generates force on actin filaments,” Trends Biochem. Sci. 41, 989–997 (2016). 10.1016/j.tibs.2016.09.006 27717739 PMC5123969

[15] M. Kapustina and C. W. Carter, Jr. , “ Computational studies of tryptophanyl-tRNA synthetase: Activation of ATP by induced-fit,” J. Mol. Biol. 362, 1159–1180 (2006). 10.1016/j.jmb.2006.06.078 16949606

[16] S. Cammer and C. W. Carter, Jr. , “ Six rossmannoid folds, including the class I aminoacyl-tRNA synthetases, share a partial core with the anticodon-binding domain of a class II aminoacyl-tRNA synthetase,” Bioinformatics 26(6), 709–714 (2010). 10.1093/bioinformatics/btq039 20130031 PMC2852213

[17] M. Kapustina , V. Weinreb , L. Li , B. Kuhlman , and C. W. Carter, Jr. , “ A conformational transition state accompanies tryptophan activation by *B. stearothermphilus* tryptophanyl-tRNA synthetase,” Structure 15, 1272–1284 (2007). 10.1016/j.str.2007.08.010 17937916 PMC2693061

[18] A. Tropsha , C. W. J. Carter , S. Cammer , and I. I. Vaisman , “ Simplicial neighborhood analysis of protein packing (SNAPP): A computational geometry approach to studying proteins,” Methods Enzymol. 374, 509–544 (2003). 10.1016/S0076-6879(03)74022-1 14696387

[19] C. W. Carter, Jr. , B. LeFebvre , S. A. Cammer , A. Tropsha , and M. H. Edgell , “ Four-body potentials reveal protein-specific correlations to stability changes caused by hydrophobic core mutations,” J. Mol. Biol. 311, 625–638 (2001). 10.1006/jmbi.2001.4906 11518520

[20] S. N. Chandrasekaran and C. W. Carter, Jr. , “ Adding torsional interaction terms to the anisotropic network model improves the PATH performance, enabling detailed comparison with experimental rate data,” Struct. Dyn. 4, 032103 (2017). 10.1063/1.4976142 28289692 PMC5315668

[21] Y. Liu and B. Kuhlman , “ RosettaDesign server for protein design,” Nucleic Acids Res. 34, W235–W238 (2006). 10.1093/nar/gkl163 16845000 PMC1538902

[22] L. Li and C. W. Carter, Jr. , “ Full implementation of the genetic code by tryptophanyl-tRNA synthetase requires intermodular coupling,” J. Biol. Chem. 288, 34736–34745 (2013). 10.1074/jbc.M113.510958 24142809 PMC3843085

[23] G. Q. Tang , J. J. Hobson , and C. W. Carter, Jr. , “ The LeuAC urzyme catalyzes aminoacylation of tRNALeu minihelix using leucine and ATP” (unpublished) (2023).

[24] P. Retailleau , X. Huang , Y. Yin , M. Hu , V. Weinreb , P. Vachette , C. Vonrhein , G. Bricogne , P. Roversi , V. Ilyin , and C. W. Carter, Jr. , “ Interconversion of ATP binding and conformational free energies by tryptophanyl-tRNA synthetase: Structures of ATP bound to open and closed, pre-transition conformations,” J. Mol. Biol. 325, 39–63 (2003). 10.1016/S0022-2836(02)01156-7 12473451

[25] V. A. Ilyin , B. Temple , M. Hu , G. Li , Y. Yin , P. Vachette , and C. W. Carter, Jr. , “ 2.9 Å crystal structure of ligand-free tryptophanyl-tRNA synthetase: Domain movements fragment the adenine nucleotide binding site,” Protein Sci. 9, 218–231 (2000). 10.1110/ps.9.2.218 10716174 PMC2144547

[26] V. Weinreb , G. Weinreb , and C. W. Carter, Jr. , “ High-throughput thermal denaturation of tryptophanyl-tRNA synthetase combinatorial mutants reveals high-order energetic coupling determinants of conformational stability,” BioRxiv (2023). 10.1063/4.0000182 PMC10449480 37637481

[27] B. Williams II , M. Convertino , J. Das , and N. V. Dokholyan , “ ApoE4-specific misfolded intermediate identified by molecular dynamics simulations,” PLoS Comput. Biol. 11(10), e1004359 (2015). 10.1371/journal.pcbi.1004359 26506597 PMC4623519

[28] N. V. Dokholyan , S. V. Buldyrev , E. Stanley , and E. I. Shakhnovich , “ Identifying the protein folding nucleus using molecular dynamics,” J. Mol. Biol. 296, 1183–1188 (2000). 10.1006/jmbi.1999.3534 10698625

[29] D. Shirvanyants , F. Ding , D. Tsao , S. Ramachandran , and N. V. Dokholyan , “ Discrete molecular dynamics: An efficient and versatile simulation method for fine protein characterization,” J. Phys. Chem. B 116, 8375–8382 (2012). 10.1021/jp2114576 22280505 PMC3406226

[30] F. Ding , D. Tsao , H. Nie , and N. V. Dokholyan , “ *Ab-initio* folding of proteins with all-atom discrete molecular dynamics,” Structure 16, 1010–1018 (2008). 10.1016/j.str.2008.03.013 18611374 PMC2533517

[31] N. V. Dokholyan , S. V. Buldyrev , E. Stanley , and E. I. Shakhnovich , “ Discrete molecular dynamics studies of the folding of a protein-like model,” Folding Des. 3, 577–587 (1998). 10.1016/S1359-0278(98)00072-8 9889167

[32] N. V. Dokholyan , L. Li , F. Ding , and E. I. Shakhnovich , “ Topological determinants of protein folding,” Proc. Natl. Acad. Sci. U. S. A. 99, 8637–8641 (2002). 10.1073/pnas.122076099 12084924 PMC124342

[33] H. C. Andersen , “ Molecular dynamics simulations at constant pressure and/or temperature,” J. Chem. Phys. 72, 2384–2393 (1980). 10.1063/1.439486

[34] T. Lazaridis and M. Karplus , “ Effective energy functions for protein structure prediction,” Curr. Opin. Struct. Biol. 10, 139–145 (2000). 10.1016/S0959-440X(00)00063-4 10753811

[35] E. Duboué-Dijon , P. E. Mason , H. E. Fischer , and P. Jungwirth , “ Hydration and ion pairing in aqueous Mg^2+^ and Zn^2+^ solutions: Force field description aided by neutron scattering experiments and ab initio molecular dynamics simulations,” J. Phys. Chem. B 122(13), 3296–3306 (2017). 10.1021/acs.jpcb.7b09612 29116789

[36] M. T. Panteva , G. M. Giambaşu , and D. M. York , “ Force field for Mg^2+^, Mn^2+^, Zn^2+^, and Cd^2+^ ions that have balanced interactions with nucleic acids,” J. Phys. Chem. B 119, 15460–−15470 (2015). 10.1021/acs.jpcb.5b10423 26583536 PMC4762653

[37] S. Kumar , D. Bouzida , R. H. Swendsen , P. A. Kollman , and J. M. Rosenberg , “ The weighted histogram analysis method for free-energy calculations on biomolecules. I. The method,” J. Comput. Chem. 13(8), 1011–1021 (1992). 10.1002/jcc.540130812

[38] W. Kabsch and C. Sander , “ Dictionary of protein secondary structure: Pattern recognition of hydrogen-bonded and geometrical features,” Biopolymers 22, 2577–2637 (1983). 10.1002/bip.360221211 6667333

[39] W. G. Touw , C. Baakman , J. Black , T. A. H. te Beek , E. Krieger , R. P. Joosten , and G. Vriend , “ A series of PDB-related databanks for everyday needs,” Nucl. Acids Res. 43, D364–D368 (2015). 10.1093/nar/gku1028 25352545 PMC4383885

[40] The Pymol Molecular Graphics System, version 2.3.1, Schrödinger, LLC, New York, NY, 2012.

[41] P. Laowanapiban , M. Kapustina , C. Vonrhein , M. Delarue , P. Koehl , and C. W. Carter, Jr. , “ Independent saturation of three TrpRS subsites generates a partially-assembled state similar to those observed in molecular simulations,” Proc. Natl. Acad. Sci. U. S. A. 106, 1790–1795 (2009). 10.1073/pnas.0812752106 19174517 PMC2644116

[42] E. V. Proctor and N. V. Dokholyan , “ Applications of discrete molecular dynamics in biology and medicine,” Curr. Opin. Struct. Biol. 37, 9–13 (2016). 10.1016/j.sbi.2015.11.001 26638022 PMC4834256

[43] F. Ding and N. V. Dokholyan , “ Simple but predictive protein models,” Trends Biotechnol. 23, 450–455 (2005). 10.1016/j.tibtech.2005.07.001 16038997

[44] V. N. Uversky and A. K. Dunker (eds.), *Intrinsically Disordered Protein Analysis: Volume 2, Methods and Experimental Tools* ( Springer Science+Business Media, New York, 2012).

[45] V. Weinreb , University of North Carolina at Chapel Hill (unpublished) (2014).

[46] J. Chen , N. Sawyer , and L. Regan , “ Protein–protein interactions: General trends in the relationship between binding affinity and interfacial buried surface area,” Protein Sci. 22(4), 510–515 (2013). 10.1002/pro.2230 23389845 PMC3610057

[47] S. Lewis , N. Yan , D. Erie , and C. W. Carter, Jr., University of North Carolina at Chapel Hill (unpublished) (2007).

[48] F. Ding , R. K. Jha , and N. V. Dokholyan , “ Scaling behavior and structure of denatured proteins,” Structure 13, 1047–1054 (2005). 10.1016/j.str.2005.04.009 16004876

[49] Y. Pham , B. Kuhlman , G. L. Butterfoss , H. Hu , V. Weinreb , and C. W. Carter, Jr. , “ Tryptophanyl-tRNA synthetase urzyme: A model to recapitulate molecular evolution and investigate intramolecular complementation,” J. Biol. Chem. 285, 38590–38601 (2010). 10.1074/jbc.M110.136911 20864539 PMC2992291

[50] P. J. Sapienza , L. Li , T. Williams , A. L. Lee , and C. W. Carter, Jr. , “ An ancestral tryptophanyl-tRNA synthetase precursor achieves high catalytic rate enhancement without ordered ground-state tertiary structures,” ACS Chem. Biol. 11, 1661–1668 (2016). 10.1021/acschembio.5b01011 27008438 PMC5461432

[51] Y. Pham , L. Li , A. Kim , O. Erdogan , V. Weinreb , G. Butterfoss , B. Kuhlman , and C. W. Carter, Jr. , “ A minimal TrpRS catalytic domain supports sense/antisense ancestry of class I and II aminoacyl-tRNA synthetases,” Mol. Cell 25, 851–862 (2007). 10.1016/j.molcel.2007.02.010 17386262

[52] V. Muñoz and L. Serrano , “ Intrinsic secondary structure propensities of the amino acids, using statistical ϕ-ψ matrices: Comparison with experimental scales,” Proteins 20(4), 301–311 (1994). 10.1002/prot.340200403 7731949

[53] K. Pervushin , K. Vamvaca , B. Vogeli , and D. Hilvert , “ Structure and dynamics of a molten globular enzyme,” Nat. Struct. Mol. Biol. 14, 1202–1206 (2007). 10.1038/nsmb1325 17994104

[54] K. Vamvaca , B. Vögeli , P. Kast , K. Pervushin , and D. Hilvert , “ An enzymatic molten globule: Efficient coupling of folding and catalysis,” Proc. Natl. Acad. Sci. U. S. A. 101, 12860–12864 (2004). 10.1073/pnas.0404109101 15322276 PMC516485

[55] C. W. Carter, Jr. , “ Coding of class I and II aminoacyl-tRNA synthetases,” in *Protein Reviews* (Springer, 2017), Vol. 18, pp. 103–148. 10.1007/5584_2017_93 PMC5927602 28828732

